# NPK macronutrients and microRNA homeostasis

**DOI:** 10.3389/fpls.2015.00451

**Published:** 2015-06-16

**Authors:** Franceli R. Kulcheski, Régis Côrrea, Igor A. Gomes, Júlio C. de Lima, Rogerio Margis

**Affiliations:** ^1^Departamento de Biofísica, Laboratório de Genomas e Populações de Plantas, Centro de Biotecnologia, Universidade Federal do Rio Grande do Sul, Porto AlegreBrazil; ^2^Departamento de Genética, Universidade Federal do Rio de Janeiro, Rio de JaneiroBrazil; ^3^Laboratório de Genética Molecular, Instituto de Ciências Biológicas, Universidade de Passo Fundo, Passo FundoBrazil

**Keywords:** nitrogen, phosphorus, potassium, microRNAs, plant nutrition, plant–microbe symbiosis

## Abstract

Macronutrients are essential elements for plant growth and development. In natural, non-cultivated systems, the availability of macronutrients is not a limiting factor of growth, due to fast recycling mechanisms. However, their availability might be an issue in modern agricultural practices, since soil has been frequently over exploited. From a crop management perspective, the nitrogen (N), phosphorus (P), and potassium (K) are three important limiting factors and therefore frequently added as fertilizers. NPK are among the nutrients that have been reported to alter post-embryonic root developmental processes and consequently, impairs crop yield. To cope with nutrients scarcity, plants have evolved several mechanisms involved in metabolic, physiological, and developmental adaptations. In this scenario, microRNAs (miRNAs) have emerged as additional key regulators of nutrients uptake and assimilation. Some studies have demonstrated the intrinsic relation between miRNAs and their targets, and how they can modulate plants to deal with the NPK availability. In this review, we focus on miRNAs and their regulation of targets involved in NPK metabolism. In general, NPK starvation is related with miRNAs that are involved in root-architectural changes and uptake activity modulation. We further show that several miRNAs were discovered to be involved in plant–microbe symbiosis during N and P uptake, and in this way we present a global view of some studies that were conducted in the last years. The integration of current knowledge about miRNA-NPK signaling may help future studies to focus in good candidates genes for the development of important tools for plant nutritional breeding.

## Introduction

Nutrients in plants have important functions in osmotic regulation, cellular permeability, and may act as structural components and essential metabolites, being therefore critical for proper growth and development. Some of those nutrients, known as macronutrients, though, are required in relatively large amounts. Among these macronutrients, nitrogen (N), phosphorus (P), and potassium (K) are three important limiting factors frequently added as fertilizers in modern agricultural schemes.

Plants have evolved several physiological and molecular adaptive responses to deal with the lack of nutrients. A better understanding about how those circuits are triggered, transduced and controlled may lead to the development of important tools for plant breeding and manipulation. Recent data have showed that classes of long and small non-coding RNAs (ncRNAs) play an important role during plant stress response (Kulcheski et al., 2011; Jeong and Green, 2013; Kumar, 2014), including in nutrient availability (Sunkar et al., 2012). The vast majority of those ncRNAs remain poorly understood, but others, like some microRNAs (miRNAs) have been extensively studied and characterized, especially as negative regulators of mRNA half-life and translation, consequently interfering with protein production.

Since miRNAs are involved in the regulation of virtually all cellular metabolic pathways, modulation of their biogenesis is of paramount importance for the maintenance of cellular homeostasis. Like mRNAs, miRNAs are mainly transcribed by RNA Polymerase II (Pol II) and are associated with factors involved in RNA splicing and processing, including addition of 5′CAP and 3′ polyadenylation (Xie et al., 2005; Bielewicz et al., 2013). Likewise, miRNA promoters have TATA box cis elements and are recognized by basic transcription initiation factors, such as transcription factor IIB (TFIIB), involved in the formation of Pol II pre-initiation complex (Megraw et al., 2006; Zhao et al., 2013). Therefore, miRNA biogenesis is temporally and spatially regulated during development, as well as in response to environmental factors. Due to their sessile nature, plants have efficiently integrated the miRNA response for the mitigation of several types of stresses.

Like other small ncRNAs, mature miRNAs are single-stranded molecules with 20–24 nucleotides (nt) in length. Those molecules, however, have a unique biogenesis mechanism. As the first step, miRNAs are produced from a particular type of stem-loop precursor RNA, termed pri-miRNAs. The pri-miRNAs are generally transcribed by RNA Pol II and may contain several 100s of nucleotides (Rogers and Chen, 2013). In eukaryotes, a special class of RNase III enzyme called Dicer recognizes and degrades pri-miRNAs, generating a stem loop intermediate known as pre-miRNA. Pre-miRNAs are further processed into 20–24 nt duplex miRNAs. In plants, the two processing steps are mediated by a single enzyme, Dicer-like 1 (DCL1; Reinhart et al., 2002; Finnegan et al., 2003; Xie et al., 2004; Park et al., 2005). DCL1 dices stem-loop RNAs into 21 nt sequences, explaining the predominance of this small RNA size class in plants. Some precursors, however, can be degraded by other plant DCLs, generating miRNAs with different sizes (Margis et al., 2006; Rajagopalan et al., 2006; Vazquez et al., 2008; Ben Amor et al., 2009).

Dicer-like 1, however, depends on several other proteins to exert its activity, forming a complex frequently refereed as microprocessor. Proteins directly or indirectly associated with DCL1 includes the dsRNA binding proteins DRB1 (also known as HYL1) and Tough (TGH; Ren et al., 2012), the zinc finger protein Serrate (SE; Yang et al., 2006), the phosphatase C-terminal Domain Phosphatase-like 1 (CPL1; Manavella et al., 2012), the threonine binding protein Dawdle (DDL; Yu et al., 2008), the proline-rich protein Sickle (SIC; Zhan et al., 2012), the RNA binding protein Modifier of SNC1-2 (MOS2; Wu et al., 2013), proteins involved in RNA splicing, like Cap Binding protein 20 (CBP20), Cap Binding protein 80 (CBP80), and Stabilized 1 (STA1), and components of the transcriptional machinery, such as Negative on TATA 2b (NOT2b; Gregory et al., 2008; Kim et al., 2008; Laubinger et al., 2008). Each protein has a specific function in the processing complex, including recruitment of pri-miRNAs, assistance in dicing activity, post-translation modification of key components and binding and/or recruitment of RNA splicing and transcriptional factors, indicating that pri-miRNA transcription and processing might occur simultaneously.

Mature duplex miRNAs may also suffer edition before being incorporated into effector complexes known as RNA-Induced Silencing Complex (RISC). Modifications may include 3′ end methylation, base additions, or degradation (Yu et al., 2005; Ramachandran and Chen, 2008). Once in RISC, duplex miRNAs binds to effector proteins called Argonaute (AGO). One of the strands is removed and the other will guide the complex to target sequences. Depending on the base pairing between the two RNAs, AGO proteins may regulate target sequences by slicing or translation inhibition (Poulsen et al., 2013). Most of known miRNA targets are mRNAs, however, other ncRNAs (Fei et al., 2013) and transposons can also be regulated during reprogramming of germ lines (Creasey et al., 2014).

Several miRNA genes/families are conserved among plants. Those genes are usually highly regulated and target mRNAs coding for development-related transcriptional factors (Willmann and Poethig, 2007). For example, genes involved with vegetative phase transition and leaf polarity are directly or indirectly regulated by conserved miRNAs (Peragine et al., 2004; Nogueira et al., 2007). Accordingly, mutations in genes associated with miRNA biogenesis display strong developmental defects and in some cases are lethal (Jacobsen et al., 1999; Lu and Fedoroff, 2000; Morel et al., 2002). However, like any other gene, miRNA genes can be continuously gained and lost during evolutionary processes. Even in closely related plants, like the two model plants *Arabidopsis thaliana* and *Arabidopsis lyrata*, about 13% of miRNA genes are unique in each species (Fahlgren et al., 2010). Most of those young miRNAs are frequently associated with clade-specific processes, including several types of stresses (Willmann and Poethig, 2007). More specifically, miRNAs have already been associated in cold, drought, salt, oxidative, injury, and nutrient stresses, among others (Sunkar et al., 2012). In fact, *drb1* and *cpb80* mutants from *Arabidopsis* are hypersensitive to the abscisic acid hormone (ABA), a key regulator of stress responses (Kim et al., 2008).

The regulation of gene expression, mediated by miRNAs in stress and development responses in plants, is enhanced by two mechanisms: amplification and migration. Depending on the size of the mature miRNA sequence, the number of binding sites to the target sequence and also the type of AGO associated, the miRNA-mediated regulation can trigger the recruitment of the amplification machinery containing the RNA-dependent RNA Polymerase 6 (RDR6; Fei et al., 2013). By creating novel dsRNAs from target-sequences, a plethora of small interfering RNAs (siRNAs) are produced by the action of DCL4 protein. Those siRNAs can also be incorporated in AGO-containing RISC complexes, creating a complex network of regulation (MacLean et al., 2010). Furthermore, both amplified siRNAs and miRNAs can move from cell-to-cell and eventually reach phloem cells, promoting a long-distance regulation (Sarkies and Miska, 2014).

These characteristics confer to miRNAs an efficient buffer capacity and, in concert with transcription factors, important hubs for both local and systemic gene regulation. In this review, we will focus on the role of miRNAs in response to the NPK macronutrient stresses, the three main growth-limiting elements that are widely used as fertilizers.

## Nitrogen and Plant miRNAs

Nitrogen is an essential macronutrient required for plant growth and development. This element is required in large amounts in plant cells, not only as an important building block of amino acids, nucleic acids, and chlorophyll, but also due the pivotal regulator role in carbon and amino acid metabolism, as well as in protein synthesis (Frink et al., 1999; Cai et al., 2012). Plants absorb N from soil in the form of nitrate (NO_3_^-^), ammonia/ammonium (NH_3_/NH_4_^+^), or urea [CO (NH_2_)_2_], and also as free amino acids or organic N through microbial symbiosis in legumes (Williams and Miller, 2001; Fischer et al., 2013). Since N is indispensable to plant growth, it is also associated with crop production improvement. In this way, millions of tons of nitrogenous fertilizers are added to the soil worldwide annually (Good et al., 2004). However this practice increases the costs of plant production and also contributes for a serious soil and water pollution due to an excess of N that remains in the environment (Good and Beatty, 2011; Fischer et al., 2013). Incomplete capture and poor conversion of N fertilizer also can causes global warming through emissions of nitrous oxide (Montzka et al., 2011). For this reason, one of the main goals of researches on plant nutrition is to improve the plant N uptake as well as its efficient use (Hirel et al., 2007).

The efficient use of N by plants includes its uptake, assimilation, translocation, and when the plant is aging, recycling, and remobilization (Masclaux-Daubresse et al., 2008; Chardon et al., 2010). Many efforts have been done to understand the molecular basis of plant responses to N and to identify N responsive genes. The complex and diverse physiological and biochemical changes involved in N metabolism suggest that a plethora of genes and metabolic pathways are necessary to allow plant adaptation according to the N presence or limitation. In this scenario, many studies comprising miRNAs responsive to N stimulus have been developed.

Several miRNAs have been characterized in association to N stresses (Pant et al., 2009; Liang et al., 2012) and are summarized in **Table 1**. Some of these miRNAs were observed to be upregulated or downregulated depending the specie, tissues, and experiment design. For example, reverse transcription quantitative real-time polymerase chain reaction (RT-qPCR) detected that some conserved miRNAs can be either repressed or induced in N-limited seedlings of *Arabidopsis* (Pant et al., 2009). The rapessed (*Brassica napus*) phloem sap was investigated about the N-responsive miRNAs, and in this species, it was observed that miR2111, miR169, and miR827-like sequences were strongly dependent on the N status (Pant et al., 2009). Liang et al. (2012), through deep sequencing technology, observed that members from the same miRNA families displayed differential expression in response to N deficiency in *Arabidopsis*. One year later, Vidal et al. (2013) studying N-responsive genes in *Arabidopsis* roots, discovered a new miRNA (miR5640) and its respective target (AtPPC3 protein) which seems to integrate the N and carbon metabolism. The AtPPC3 protein is one of the four phosphoenolpyruvate carboxylase isozymes and is involved in the carbon metabolism that catalyzes the β-carboxylation of phosphoenolpyruvate to yield oxaloacetate. In C3 plants and algae, AtPPCs are important for the production of carbon skeletons used for N assimilation. Despite, the missing information about AtPPC3 function in N metabolism, Vidal et al. (2013) observed that this protein was nitrate-induced, being a good candidate for further investigation.

**Table 1:** N-responsive miRNAs with respective expression profiles according to different treatments, tissues, and species.

Plant species	miRNAs	Expression profile	Samples/tissue	N condition	Reference
*Arabidopsis thaliana*	miR156e, miR156g, miR157d	Upregulated	Seedlings	N-deficient	Pant et al. (2009)
	miR169h, miR169i, miR169j, miR169k, miR169l, miR169m, miR169n, miR398	Downregulated			
	miR160, miR780, miR826, miR842, miR846	Upregulated	Whole seedlings	N-deficient	Liang et al. (2012)
	miR169, miR171, miR395, miR397, miR398, miR399, miR408, miR827, miR857	Downregulated			
	miR5640	Downregulated	Roots	1 h after N addition	Vidal et al. (2013)
*Brassica napus*	miR156, miR399	Upregulated	Phloem sap	N-deficient	Pant et al. (2009)
	miR159, miR169, miR2111	Downregulated			
*Glycine max*	miR172l-3p, miR396bcdfg-5p, miR396bcg-3p, miR398ab-3p, miR1511-3p, miR4413a-5p	Upregulated	Roots	N-deficient (short-term treatment)	Wang et al. (2013)*
	miR156p-5p, miR171n-3p, miR171o-5p, miR390ac-3p, miR482a-3p, miR4348-5p	Downregulated			
*Oryza sativa*	miR156, miR164, miR528, miR820, miR821, miR1318	Downregulated	Leaves	N-deficient (low-N tolerante/low-N sensitive genotype)	Nischal et al. (2012)
	miR164, miR167, miR168, miR528	Downregulated	Roots		
*Phaseolus vulgaris*	miR167, miR169, miR319, miR399, miR408	Downregulated	Leaves/roots	N-deficient	Valdés-López et al. (2010)
	miR396	Upregulated	Leaves		
*Populus tomentosa*	miR393, miR395,miR396abe	Upregulated	Whole plantlets	N-deficient	Ren et al. (2015)
	miR159, miR160, miR162, miR166, miR167, miR168, miR169, miR171, miR172, miR390, miR396c, miR399, miR403, miR475, miR482, miR1448, miR6427, miR6445, miR6462	Downregulated			
*Zea mays*	miR167, miR169, miR395, miR399, miR408, miR528	Downregulated	Roots	Long term N-deficient	Xu et al. (2011)
	miR164, miR172, miR827	Upregulated	Leaves		
	miR169, miR397, miR398, miR399, miR498, miR528	Downregulated			
	miR160, miR168, miR169, miR319, miR395, miR399	Upregulated	Roots	Short-term N-deficient	
	miR172	Upregulated	Leaves		
	miR397, miR398, miR827	Downregulated			
	miR162, miR167, miR394	Upregulated	Shoots	N-deficient	Zhao et al. (2012)
	miR169, miR397, miR398, miR408, miR528s	Downregulated			
	miR162, miR167s	Upregulated	Roots		
	miR169, miR169cr, miR395abdefghijnp, miR397s, miR408s, miR528s, miR395s, miR827	Downregulated			
	miR166jkn, miR169ijk, miR408b, miR528ab	Downregulated	Roots	N-deficient	Trevisan et al. (2012a,b)

*These data are from RT-qPCR analysis, this paper also presents a huge set of miRNAs profile from deep sequencing analysis.

In maize, Xu et al. (2011) studied a detailed response of miRNAs in shoots and roots under long-term and short-term low N condition. The results were interesting, since they showed some miRNAs having different behavior across the different N starvation kinetic, like was the case of miR169 in roots. However, the other high conserved miRNA, miR172 presented the same profile in leaves in both experimental conditions. Investigating the molecular biology of miRNAs underlying N sensing/signaling in this same crop, Zhao et al. (2012) constructed four small RNA libraries from shoot and root under N-sufficiency and deficiency. The sequencing data analysis showed that in shoots and roots the expression of some conserved miRNAs was variable under N deficiency. Also, in this study, the authors showed that RT-qPCR and small RNA northern blot results are consistent with the results obtained by sequencing, which is not a rule, once in several cases, discrepancies are observed across those methodologies. Trevisan et al. (2012a,b) also observed the N effect on miR528ab, miR169ijk, miR166jkn, and miR408b transcripts accumulation. Since they employed the *in situ* hybridization methodology, the inspection of tissue localization of these miRNAs was possible. All miRNAs analyzed showed transcript accumulation in N-supplied roots, while signals became weaker in N-depleted roots. In N-supplied roots, miR169ijk, and miR166jkn were present exclusively in the root vascular tissues, miR528ab in pericycle cells and miR408b in epidermis tissue. However miR169ijk was only found in nitrate-supplied root tips, while the others miRNA were detected also in N-deficient roots.

Rice is the other important crop which has being investigated about miRNAs expression under N stresses. Nischal et al. (2012) investigated microarray-based miRNA expression in N-tolerant and N-sensitive rice genotypes under low N condition. They detected the expression of some miRNAs and their respective targets. In both tissues analyzed, miRNAs were downregulated and their respective targets expressions were significantly higher in low N-tolerant genotype than low N-sensitive genotype. The majority of miRNAs targets identified were transcription factors and proteins associated with metabolic processes or stress responses. In soybean, Wang et al. (2013) performed a massive sequencing of 16 different libraries. Using the sequencing data, they found that multiple members of the miR169 family were repressed in both roots and shoots of two soybean varieties under low N stress. Other conserved miRNA family, the miR156, also showed a variable response according the different experimental parameters. Aside from the sequencing data, the authors also performed RT-qPCR to predict some miRNAs expression (data showed in **Table 1**). Previous study exploring the miRNA expression in other legume, the blackbean (*Phaseolus vulgaris*), also detected the repression of some conserved in leaves and roots during N starvation; being the miR396 the unique conserved miRNA upregulated in leaves tissues (Valdés-López et al., 2010).

Recently, the Chinese tree, *Populus tomentosa*, was investigated about its responsive miRNAs to N stress. In this study Ren et al. (2015) identified conserved and new miRNAs, and also employed RT-qPCR to determine the miRNAs dynamic and responses to low N stress at different time points. In general, they observed a variable behavior across the miRNAs families with a consistency among the members of each family. The family miR396 was the unique to present an inconsistency variation among their members, for example, miR396a, miR396b, and miR396e were upregulated in response to low N stress, but miR396c was downregulated.

**FIGURE 1:**
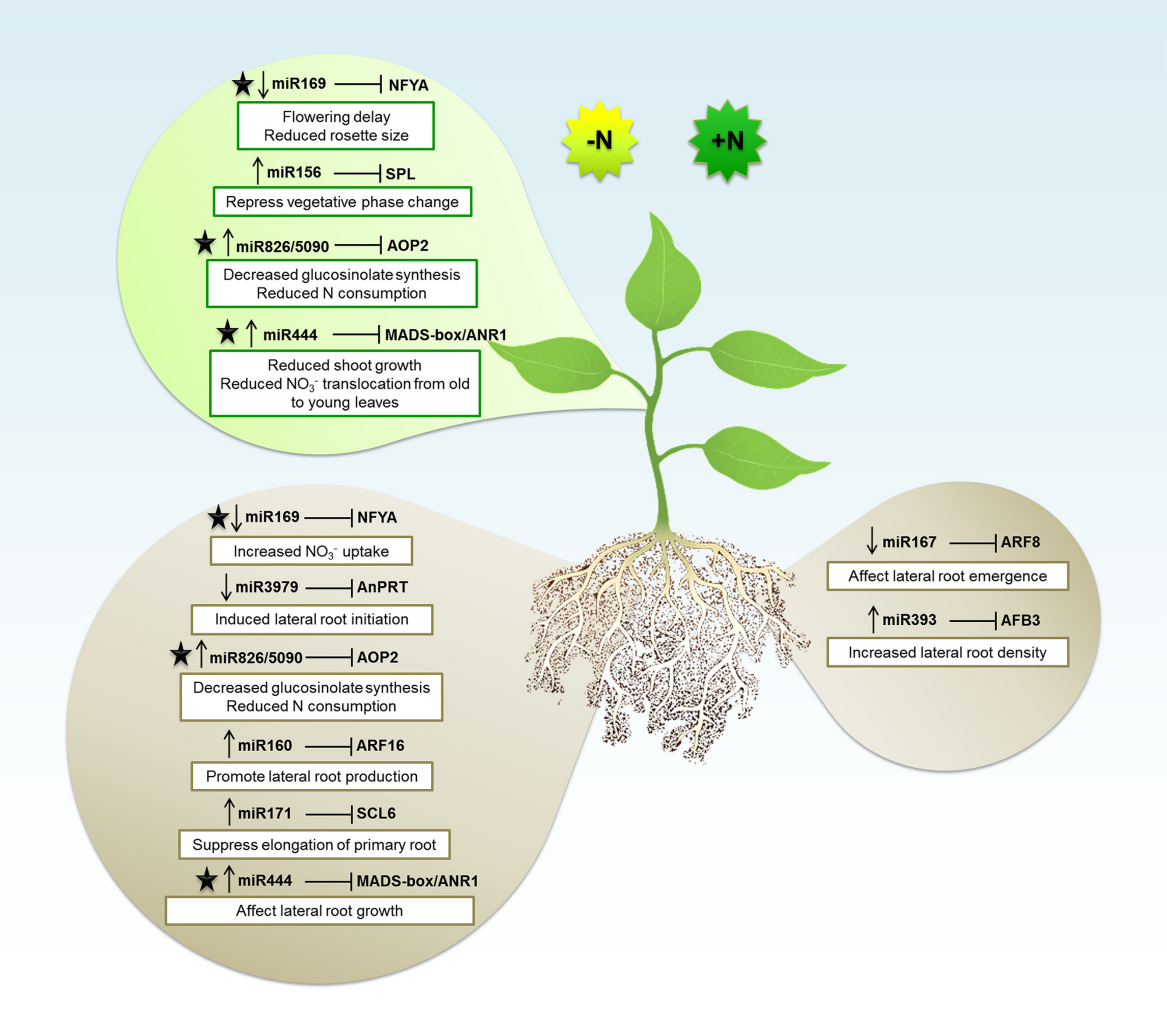
**The microRNA (miRNA) regulatory network in Nitrogen (N) signaling**. The miRNAs expression patterns during N-deficient (-N) or N-sufficient (+N) in shoot (top balloon) or root (bottom balloons) tissues. Arrows pointing down and up indicate downregulation and upregulation, respectively. Each miRNA is associated with the mRNA target gene that is inhibited. Depending of the miRNA regulation, the target will be induced or repressed which will consequently affect plant structures and biological processes (described in white boxes). The black star indicates the miRNAs and respective targets that were explored about N signaling in both root and shoot tissues.

Taking all these published work that were summarized above, as well as in **Table 1**, we can observe that in general the miRNAs tend to be downregulated during N deficiency. However, there are lots of variations according to different species, tissues, and experimental conditions that can affect a miRNA expression and results in an unstable behavior. Usually in these studies, the expression analyses are done in a high throughput way providing a huge amount of data. Global analyses are very helpful once they supply a plethora of possible miRNAs that are involved in particular process, facilitating the selection of potential candidates to be further explored. So, on the next sections we will discuss about some miRNAs, as also their respective targets, that have been deeply analyzed and experimentally confirmed to be involved in N response and symbiotic N fixation. The results of these studies are summarized in **Figure 1**.

### The Nitrogen Responsive miRNAs and Their Targets

#### miR156 and Squamosa Promoter Binding Protein Like (SPL)

The miR156 is one of the most conserved and ubiquitous miRNAs in plants and is conserved throughout the plant kingdom (Axtell and Bowman, 2008). In *Arabidopsis*, miR156 targets 11 out of the 17 members from the SPL gene family (Rhoades et al., 2002; Xing et al., 2010; Gou et al., 2011). The miR156 and its SPLs targets define an essential regulatory module that controls phase transitions, leaf trichome development, male fertility, embryonic patterning, and anthocyanin biosynthesis (Wang et al., 2009a, 2014b; Nodine and Bartel, 2010; Xing et al., 2010; Yu et al., 2010). SPL proteins play critical roles in maintaining normal growth throughout plant life cycle. These miRNAs are involved in phase change via their targets, members of the SPL transcription factors. The miR156a and miR156c levels have been shown to be repressed by a factor produced by leaf primordia, as defoliation generates high levels of miR156 (Yang et al., 2011). Recent work showed that the leaf-derived signal might be glucose or a glucose-derived metabolite, since glucose represses the expression of miR156 genes promoting vegetative phase change (Yang et al., 2013; Yu et al., 2013). These results suggest that products of photosynthesis can act as positive signals for the plant to proceed into the adult phase. Similarly, N limitation can induce the expression of miR156 in *Arabidopsis* seedlings (Pant et al., 2009; Liang et al., 2012; Fischer et al., 2013). Analysis of the transcriptome of N-limited plants shows that one of the miR156 targets, SPL3, is downregulated (Krapp et al., 2012), suggesting that a miR156/SPL3 module might act by repressing vegetative phase change under limiting N availability. Furthermore, miR156 acts as a negative regulator of miR172 by controlling miR172 expression via its targets SPL9 and SPL15 (Wu et al., 2009a). Consistently, N starvation represses miR172 in *Arabidopsis* leaves (Liang et al., 2012; Fischer et al., 2013). However, no changes in transcript levels of miR172 targets are induced by N starvation in *Arabidopsis* leaves (Krapp et al., 2012).

#### miR160 and Auxin Response Factor 16 (ARF16)

In a study performed with *Arabidopsis*, was demonstrated that the abundance of miR160 was strongly increased during N-deficient compared with N-sufficient conditions (Liang et al., 2012). In this study, the authors observed that the expression induction of miR160a was co-related with the downregulation of its ARFs targets under N-deficient conditions. ARFs are DNA binding proteins that control auxin-regulated transcription and are only present in plants. They bind to auxin-responsive promoter elements, which are found in early auxin responsive genes. Some previous studies showed that miR160 regulates the number of lateral root by controlling ARF16, being this auxin responsive factor already characterized to control root cap formation (Wang et al., 2005). Investigating whether the accumulation of miR160 facilitates lateral root formation during N-deficiency, Liang et al. (2012) produced transgenic plants overexpressing miR160a. As expected by the authors, the miR160 overexpressing mutants had higher number of lateral roots than control plants, once that N-deficiency induces expression of miR160, which increases ARF16 degradation and consequently supports lateral root formation.

#### miR167 and Auxin Response Factor 8 (ARF8)

The complex process of adventitious rooting is controlled by several endogenous and environmental factors. Some years ago, Gutierrez et al. (2012) demonstrated that the auxin response factors ARF6 and ARF8, targets of the miR167, are involved in the induction of adventitious rooting. Once that is already known that N concentration can stimulate lateral root elongation (Zhang and Forde, 1998; Lopez-Bucio et al., 2003), Gifford et al. (2008) decided to investigate the role of miR167 and ARF8 in response to N stimulus in *Arabidopsis*. Analyzing how tissues respond separately and coordinate their responses to N signals, the authors looked for different gene expression in a range of cell types from inner to outer layers of root tissues. They found that ARF8 was highly expressed in the pericycle and the lateral root cap cells during N treatment, while its miRNA regulator was repressed. In wild type phenotypes, N causes an increase ratio of initiation versus emergence of lateral roots. According to Gifford et al. (2008), this is a strategy that plants employ to control root architecture under N variations. If the environment is rich in N signal, the lateral root formation is initiated; however, the stimulus for lateral root outgrowth only occurs under N-starvation. Additionally, these authors observed that transgenic *Arabidopsis* overexpressing miR167 and null mutants for *arf8* exhibited a complete loss of N control over lateral root emergence. These finds showed that the cell-specific adjustment in ARF8 by miR167 transcripts can lead to the lateral root emergence regulation as a strategic response to N influx.

#### miR169 and Nuclear Factor Y Subunit A (NFYA)

In *Arabidopsis*, members from the transcription factors family NFYA also called heme-activated protein (HAP) or CCAAT-box binding-factor (CBF), were characterized to be regulated by miR169 (Jones-Rhoades and Bartel, 2004; Li et al., 2008). Plants NFYA have being associated with nodule differentiation and drought tolerance (Combier et al., 2006). During N starvation, was observed that three members of this family, NFYA3, NFYA5, and NFYA8, are strongly induced in shoots and roots, while their repressor miR169 is suppressed (Zhao et al., 2011). Nutrient deficiency, like N, can trigger oxidative stresses signals and the NFYA5 was described to modulate downstream genes involved in oxidative stress responses, such as those encoding a subunit of the cytochrome b6-f complex, glutathione S-transferases (GST), peroxidases and an oxidoreductase family protein (Li et al., 2008). In a previous study performed by Wenkel et al. (2006), the overexpression of NFYA (or HAP2) was associated with flowering delay due a reduction of the florigen Flowering locus T (FT) levels. Some years later, a study evaluating several miRNAs and their respective targets through mimicry assays, validated the miR169 and NFYA (HAP2), and also showed that the downregulation of miR169 caused reduced rosette size in *Arabidopsis* plants (Todesco et al., 2010). In the study performed by Zhao et al. (2011), they showed that miR169 was critical for the N-starvation response in *Arabidopsis*. Analyzing transgenic *Arabidopsis* plants overexpressing miR169a, they showed a decrease in N accumulation. These plants showed higher sensitivity to N limitation comparing to the wild type, showing leaf yellowing, which can be related to the impaired capacity of N-absorption since NFYA regulates the nitrate transporters NRT1 and NRT2 (Zhao et al., 2011). Interestingly, miR169 was also reported to be N responsive in maize and soybean (Xu et al., 2011; Liang et al., 2012; Trevisan et al., 2012b; Zhao et al., 2012; Wang et al., 2013; Zeng et al., 2014).

#### miR171 and Scarecrow-Like 6 (SCL6)

The SCL transcripts are already characterized to be targeted by miR171 (Wang et al., 2010). This miRNA was associated with primary root elongation decrease through cleavage of three SCL6 transcripts (Wang et al., 2010). Quantitative RT-PCR analyses demonstrated that the miR171c expression was threefold higher under N-deficient than under N-sufficient conditions. Consistently, the three miR171 targets were downregulated at the same conditions. These results point to a miR171 induction promoted by N-starvation, which outcomes with a inhibitory effect over their targets (SCL6-II, SCL6-III, and SCL6-IV), and consequently repress the elongation of primary roots during this stress condition (Liang et al., 2012).

#### miR393 and Auxin Signaling F-Box Protein 3 (AFB3)

To investigate the small RNAs (sRNAs) role, Vidal et al. (2010) performed high throughput sequencing from *Arabidopsis* seedling submitted or not to N treatment. The authors observed that miR393 was induced by nitrate and also detected some target transcripts for this miRNA: a basic helix-loop-helix (bHLH) transcription factor and the auxin receptors TIR1, AFB1, AFB2, and AFB3. However, from all these miR393 known targets, only AFB3 was regulated by nitrate in roots under the experimental conditions. They showed that nitrate is able to transcriptionally induce expression of AFB3 in roots and that N metabolites produced after nitrate reduction and assimilation lead to a downregulation of AFB3 levels due to miR393 induction. This regulatory module, revealed an incoherent feed-forward mechanism that is induced by nitrate and repressed by N metabolites generated by nitrate reduction and assimilation. The observed regulation of AFB3 expression by nitrate and metabolites produced downstream of nitrate reduction might constitute a mechanism to rapidly and precisely adjust root growth depending on external and internal nitrate availability. As several miRNA targets encode transcription factors in plants, incoherent feed-forward loops are probably also a common feature of plant gene networks (Vidal et al., 2010). The rapid downregulation of AFB3 by miR393 provides a fine-tuned mechanism of the root system to dynamically respond to N in real time. In this same work, Vidal et al. (2010) investigated the miR393/ARF3 pathway analyzing the root architecture of ARF3 mutants and miR393 overexpressors, and they observed that these plants had primary and lateral roots growth unresponsive to N stimulus. With these findings, the authors conclude that miR393/ARF3 is the responsible mechanism to repress primary root elongation and induce lateral root emergence under the presence of N. This interaction is an excellent example about how a small regulatory molecule integrates nitrate availability with auxin signaling.

#### miR444 and MADS-Box Transcription Factor

The monocot specific miR444 has been demonstrated to regulate four MIKC-type MADS-box transcriptional factor genes in rice (OsMADS23, OsMADS27a, OsMADS27b, and OsMADS57; Sunkar et al., 2005; Lu et al., 2008; Wu et al., 2009b; Li et al., 2010b; Yan et al., 2014). Phylogenetic analysis grouped the miR444 targets with *Arabidopsis* ANR1clade (Lee et al., 2003; Arora et al., 2007), which is a pivotal regulator in NO_3_^-^ signaling pathway in lateral root growth (Zhang and Forde, 1998). Yan et al. (2014) showed that the miR444a regulates NO_3_^-^ signaling in rice root growth and nitrate accumulation. Plants overexpressing this miRNA presented a decrease in the expression of the four MADS-box genes, and a reduced nitrate induced lateral root growth. These plants also showed altered primary and adventitious root architecture in response to different nitrate concentrations, indicating that miR444a participates in the NO_3_^-^ signaling pathway through ANR1-homologous genes in rice. Additionally, overexpression of this miRNA caused a shoot decrease at the early seedling stage; as well as a higher NO_3_^-^ level in rice shoots and roots under sufficient nitrate amount. An impaired N remobilization from old to young leaves was observed in these transgenic plants under N-limiting conditions, suggesting that miR444a participates in nitrate translocation in shoots under N-starvation conditions. So far, this work showed the crucial role of miR444 in NO_3_^-^ signaling for root and shoots development and nitrate accumulation in rice.

#### miR3979 and Anthranilate Phosphoribosyl-Transferase (AnPRT)

The rice-specific miR3979 was reported to be preferentially expressed in roots tissues (Jeong et al., 2011; Jeong and Green, 2013). Once roots are responsible for N absorption, this miRNAs has being investigated about its regulation under N stress. Jeong et al. (2011) observed that miR3979 is downregulated on roots during N starvation. This miRNA was predicted to target a transcript encoding an AnPRT which is involved in tryptophan (Trp) biosynthesis. According to Zhao et al. (1998), Trp biosynthetic genes are generally induced by amino acid starvation as well as abiotic and biotic stresses. Besides to its role in protein biosynthesis, Trp is also involved on the production of secondary metabolites like the phytohormone auxin and the phytoalexin camalexin (Kutchan, 1995; Radwanski and Last, 1995; Jeong and Green, 2013). Once that auxin is involved in lateral root formation, the upregulation of Trp expression due the repression of miR3979 can be one of the regulatory mechanisms by which plants triggers lateral root initiation during N limitation.

#### miR826/miR5090 and Alkenyl Hydroxalkyl Producing 2 (AOP2)

Other miRNA characterized as N-responsive is the miR826 which was described to be upregulated during N depletion (Liang et al., 2012). The miR826 targets the AOP2 gene, which encodes a 2-oxoglutarate-dependent dioxygenase, involved in glucosinolate biosynthesis. The data generated by high throughput sequencing showed an expressive induction of miR826 in roots and shoots by N starvation, associated with significant repression of its target, AOP2. Glucosinolates are a class of plant secondary metabolites rich in N and sulfur mainly found in *Brassicaceae*. For this reason, the suppression of AOP2 by miR826 could reduce the glucosinolates biosynthesis and consequently decrease the demand for N. Two years later, He et al. (2014) detected a new miRNA, miR5090, from the complementary transcript of the miR826 gene. Similar to miR826, miR5090 is also induced by N deficiency, and both miRNAs target AOP2. To further prove the AOP2 is regulated by these miRNAs, an experiment overexpressing both miRNAs and the target were performed. The authors observed that AOP2 mRNA levels decreased considerably when coexpressed with both miRNAs compared to the control level. To confirm the cleavage site of AOP2, synonymous substitutions were introduced to generate miR826- and miR5090-resistant versions of AOP2. In this way, miR826 or miR5090 were coexpressed with the resistant version of AOP2, resulting in an unaffected AOP2 mRNA level. These data confirmed that AOP2 is the common target of miR826 and miR5090. In this same work, the authors also observed that AOP2 transcript level was negatively correlated with miR826 and miR5090 under N deficiency, corroborating with results observed by Liang et al. (2012). Besides of low AOP2 expression, the transgenic plants overexpressing miR826/5090 also accumulated fewer Met-derived glucosinolates, phenocopying the *aop2* mutants. Plants inhibit the expression of several glucosinolate synthesis genes as a mechanism to avoid the N consumption during N scarcity. So, in this work, He et al. (2014) showed that miRNA transgenic plants with less glucosinolate displayed enhanced tolerance to N starvation, including high biomass, more lateral roots, increased chlorophyll, and decreased anthocyanin. With this study was possible to figure out that the miR826/5090 – AOP2 regulatory system is involved in the plant adaptation to N-limited environment. Since AOP2 is a key enzyme in glucosinolate synthesis, its downregulation during N starvation decreases this pathway, allowing the use of N for other metabolites biosynthesis, like products necessary for plant growth and development during N limitation.

### miRNAs and Symbiotic N Fixation

Plants have evolved several strategies to improve the nutrients uptake with the help of beneficial soil microorganisms, known as N-fixing bacteria. They are the agents of the biological N fixation, which consists to reduce dinitrogen into NH_4_^+^ that is subsequently assimilated by the host plant. One of the best studied symbioses involves plant legumes and bacteria, collectively known as rhizobia. These partners cooperate in a N-fixing symbiosis of major ecological importance that occurs on all continents and accounts for a fourth of the N fixed annually on earth (Masson-Boivin et al., 2009). In roots of host plants, the interaction starts with a chemical signaling between partners, which will be necessary to confirm host specificity. The symbiosis establishment involves several molecular signals, like plant flavonoid compounds (which will be recognized by compatible rhizobia species) and bacterial lipochitooligosaccharide which are known as Nod factors. An extensive review about legumes and rhizobium signal molecules involved in the interaction was published by Janczarek et al. (2015). The detection of Nod signals and the colonization of root hairs by rhizobia cells will trigger several root alteration with a consequent development of structures called infection threads. Concomitantly, several divisions occur in cortex and pericycle cells producing the nodule primordium. Infection threads transport rhizobia into the developing nodules, where they differentiate into bacteroides and fix N. Finally, symplastic and vascular connections are formed promoting transport of nutrients to and from the mature nodules (Murray, 2011; Turner et al., 2013). Aside from their roles in N-sensing signaling, miRNAs are also being characterized in plant–microbe symbiosis. Extensive studies investigating miRNAs during symbiosis are being developed with the legumes *Glycine max* and *Medicago truncatula*.

One of the earliest studies involving miRNAs and N symbiotic fixation was performed by Subramanian et al. (2008). They investigated the potential miRNAs regulators of the earliest stages of nodule development under *Bradyrhizobium japonicum* inoculation. A kinetic evaluating three points after inoculation was carried out. The authors found some miRNAs, like miR168, and miR172 which were upregulated at the first hours and after decreased at a basal level. They found that miR159 and miR393 were continually induced, while miR160 and miR169 were downregulated during the response to rhizobia. Interestingly, two of those miRNAs, miR393, and miR160, were already identified in plant–pathogen interactions and seem to be involved in plant basal immunity promotion (Subramanian et al., 2008; Simon et al., 2009). In a related study, Wang et al. (2009b) investigating soybean nodules harvested 28 days post inoculation with *B. japonicum* detected four miRNA families (miR1507, miR1508, miR1509, and miR1510) in common to Subramanian et al. (2008) study. They also examined the expression of some miRNAs in nodule tissues. The miR172 and miR2107 were significantly upregulated in N-fixing nodules, while downregulation of miR396, miR1508, and miR1509 became evident in the nodules. Consistent with many previous studies, the majority of predicted targets detected by Wang et al. (2009b) are transcription factors, which are involved in hormone signaling and plant defense responses. Posterior studies have also corroborated the pioneer’s works involving soybean nodulation. Barros-Carvalho et al. (2014) detected the presence of miRNAs miR1530, miR1520, and miR1522 in soybean root inoculated with *B. japonicum*. These miRNAs were, previously identified by Subramanian et al. (2008) and are involved in the early stages of nodulation. Other miRNA identified in this tissue was miR4393, early identified by Joshi et al. (2010). Interestingly, besides of some conserved miRNAs which are widely detected in plant genomes, the majority of miRNAs detected in these studies were *Fabaceae* specific miRNAs, like miR1507 to miR1510, or soybean species-specific as miR1520, miR1522, miR1530, miR2107, and miR4393. The *G. max* and *M. truncatula* were already characterized to have high proportion of species-specific miRNA genes (Cuperus et al., 2011). Lelandais-Briere et al. (2009) also detected *M. truncatula* specific miRNAs, like miR2586. *In situ* analysis demonstrated that this miRNA accumulated in the nodule meristem. This is very interesting, once species-specific miRNAs are developed later during plant evolution, these miRNAs could have evolved in the symbiotic interaction pathways.

Recent works are going deeper in understanding of how miRNAs can affect soybean nodulation. Turner et al. (2013) showed that ectopic expression of miR160 resulted in a decrease in nodulation. The authors overexpressed the miR160, which regulates the ARF10/16/17 (as discussed before), resulting in a silencing of a set of repressor auxin response factor transcription factors. These plants presented root hypersensitive to auxin and had significantly reduced nodule primordium formation. On the other hand, elevated expression of miR482, miR1512, and miR1515 caused increased nodulation in soybean (Li et al., 2010a). Opposite roles have been described for miR156 and miR172 in controlling the expression of both symbiotic and non-symbiotic hemoglobins to modulate the extent of nodulation in soybean, with enhanced levels of miR156 being consistent with reduced nodule numbers while miR172 acting as a positive regulator of nodule formation (Yan et al., 2013). One of the last discoveries involving the soybean miRNA172 and nodule regulation was done by Wang et al. (2014a) and proved that the miR172c modulates both rhizobium infection and nodule organogenesis. This miRNA was induced in soybean roots inoculated with either compatible *B. japonicum* or lipooligosaccharide Nod factor and was highly upregulated during nodule development. Reduced activity and overexpression of miR172c caused dramatic changes in nodule initiation and nodule number. In this way, miR172c regulates nodule formation by repressing its target gene, Nodule Number Control1, which encodes a protein that directly targets the promoter of the early nodulin gene, ENOD40. Interestingly, transcriptional levels of miR172c were regulated by both Nod Factor Receptor1α/5α-mediated activation and by auto regulation of nodulation-mediated inhibition.

*Medicago truncatula* is a model organism broadly employed in genetic legume studies. Combier et al. (2006) explored this species to investigate the miR169 role during nodule development. As earlier demonstrated, miR169 targets the HAP2 transcription factor (Jones-Rhoades and Bartel, 2004), which was observed to be significantly induced in during symbiotic interactions (Combier et al., 2006). To prove the participation of miR169 and HAP2 during nodulation, these authors performed experiments involving miR169 overexpression and HAP2 silencing by RNAi. The miR169 overexpression caused a repression of HAP2 gene resulting in a deficient N-fixation phenotype. The RNAi assay also showed a delay in nodule development being associated with a consequent inability of N_2_ fixation. Additionally, the HAP2 expression was restricted to the nodule meristematic zone, indicating that miR169 is controlling the spatial regulation of HAP2 during this stage in N-fixing cell (Combier et al., 2006; Simon et al., 2009).

Other conserved miRNAs, like miR166 and miR396, have also being investigated in nodule development and symbiosis interactions in *M. truncatula*. The miR166 was observed to have a similar spatial expression of its target, a class III HD-ZIP transcription factor (Boualem et al., 2008). Overexpression of this miRNA leads to a reduction of HD-ZIP transcripts causing alterations in roots vascular bundle patterning and decreasing lateral root and nodule formation (Boualem et al., 2008; Simon et al., 2009). Similarly, miR396 was observed to be expressed in roots, with a differential pattern during lateral root and nodule formation (Bazin et al., 2013). It’s known that this miRNA regulates a growth-regulating factor gene (GRF) and was proved to limit mycorrhizal colonization (Bazin et al., 2013). However, experiments overexpressing miR396 or inactivating it by mimicry didn’t affect nodule density, morphology, or cellular organization after inoculation with the symbiotic bacteria *Sinorhizobium meliloti*. This scenario shows how important is to perform a deep investigation about a miRNA role, once is not possible to predict its function based only on a miRNA and its target expression. Although, both miRNA396 and GRF target are expressed in mature nodules, seems that they are not involved in the nodulation process.

A third species of legume was investigated about miRNAs and symbiotic N fixation is *Lotus japonicus*. De Luis et al. (2012) identified two miRNAs responsive to symbiotic infection and nodule function. The authors observed that the induction of a non-canonical miR171 isoform, which targets the key nodulation transcription factor Nodulation Signaling Pathway 2, correlates with bacterial infection in nodules. These finds were very interesting, since the conserved miR171 target a SCL gene family, showing the importance to explore the miRNA isoforms which can regulate alternative targets and provide different roles in plant biology. The second miRNA analyzed was the miR397, which is systemically induced in the presence of active N-fixing nodules but not in non-infected or inactive nodule organs. This miRNA, which targets a member of the laccase copper protein family, is involved in N fixation-related copper homeostasis, being a link between two different nutrient metabolisms.

## Phosphorus and Plant miRNAs

Phosphorus is an essential macronutrient required for plant growth, development, and propagation. The element corresponds to about 0.2% of plants dry weight and is a component of important macromolecules, being involved in the energetic metabolism and in transduction cell signaling pathways (Kuo and Chiou, 2011). The type of phosphate that can be directly assimilated by plants (orthophosphate or Pi) is rather scarce in the soil due to its precipitation with cations and decomposition. These factors make P one of the less available macronutrients to plants. These, in turn, led to the development of several adaptations designed to surpass such deprivation (Raghothama, 1999).

The uptake and assimilation of P in plants is orchestrated by an intricate network of proteins and tissues. Two proteins are known to play key roles in the process: phosphate transporter 1 (PHT1) and phosphate 1 (PHO1). PHT1 composes a family of transmembrane transport proteins expressed in roots and other plant tissues (Mudge et al., 2002). PHT1 proteins are able to use energy to cotransport Pi and H^+^ and are therefore involved in Pi acquisition (Okumura et al., 1998). In *Arabidopsis*, there are at least nine genes (thirteen in *Oryza sativa*) encoding PHT1 (Okumura et al., 1998; Mudge et al., 2002) and Pi deprivation stimulates the expression of most of these genes, whose products are located in the plasma membrane of cortical and epidermal root cells, indicating their involvement in nutrient uptake (Karthikeyan et al., 2002). PHO1, on the other hand, is involved in the loading of acquired Pi into xylem, facilitating therefore the root-to-shoot transport of this macronutrient in plant (Poirier et al., 1991; Hamburger et al., 2002).

Upon Pi stress, plants trigger the expression of several homeostatic mechanisms called *Pi Starvation Responses* (PSR; Raghothama, 1999). It is believed that around 900–3000 genes are involved in these responses. Since, PHT1 and PHO1 proteins are central in the assimilation and allocation of Pi in plants, PSR try to maximize their expression through several direct or indirect pathways, including post-transcriptional regulation by miRNAs. The MYB transcription factors Phosphate Starvation Regulator 1 (PHR1) and Phosphate Starvation Regulator 1-like (PHR1-LIKE1) have an important function in PSR by initiating a cascade regulation that ultimately leads to PHT1 and PHO1 over-accumulation. This function is mediated by directly or indirectly inducing their expression or by indirectly inhibiting their repressors. For example, PHR1 and PHR1-LIKE1 induce the expression of Phosphate Transporter Traffic Facilitator 1 (PHF1), a protein that facilitates the transport of PHT1 to membranes, increasing therefore their availability for Pi assimilation (Gonzalez et al., 2005; Hsieh et al., 2009; Bayle et al., 2011). In *phf1 Arabidopsis* mutant plants, PHT1 is retained in the endoplasmic reticulum, being less available in membranes (Gonzalez et al., 2005; Bayle et al., 2011). Many genes associated to Pi starvation are also constitutively expressed in *phf1* mutants, indicating the importance of PHF1 in the response regulation. At the same time, PHR1 and PHR1-LIKE1 may also trigger the expression of two miRNAs: miR399 and miR827.

**FIGURE 2:**
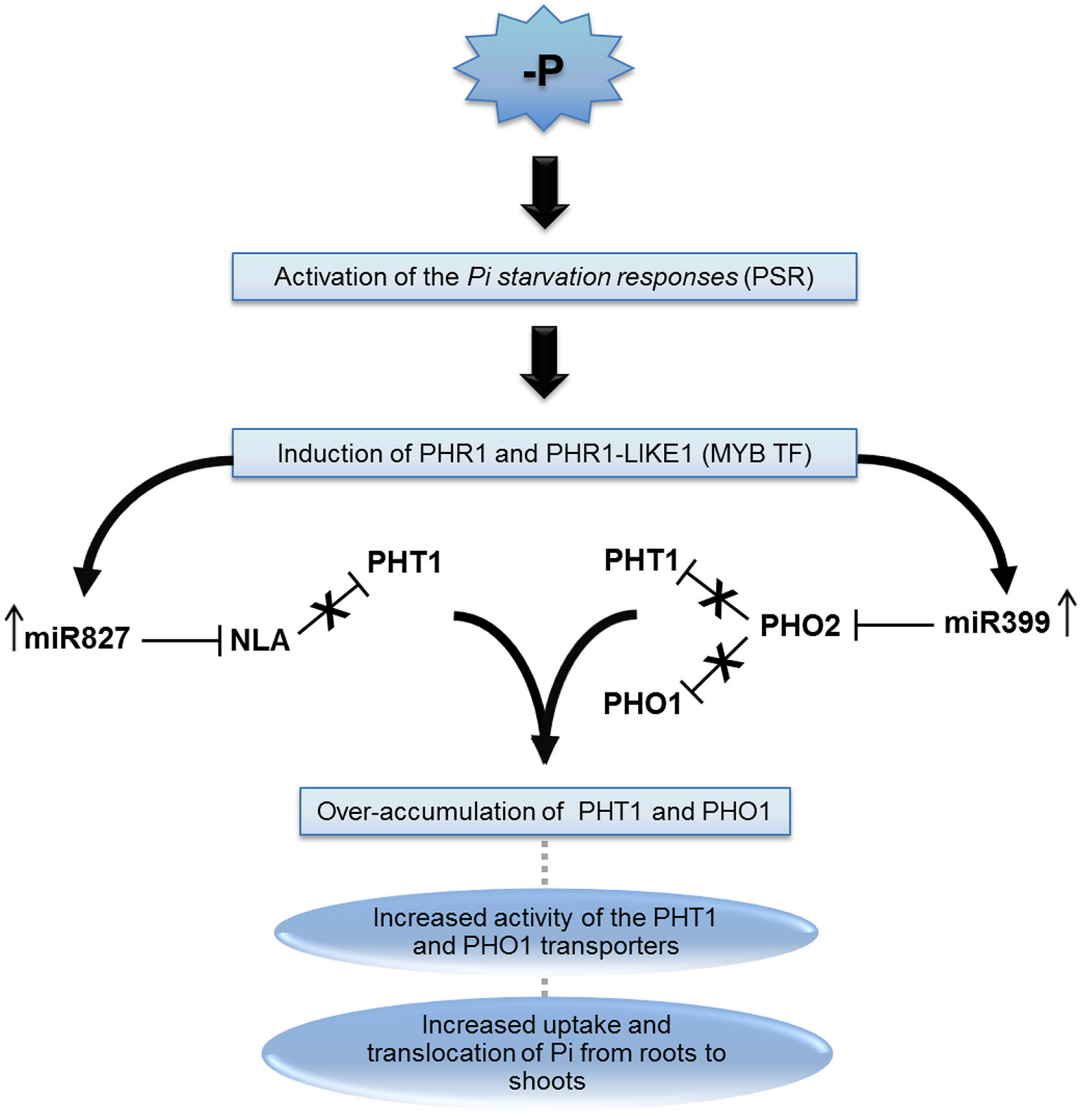
**The miRNA399 and 827 pathway involved in plant P-deficiency**. Pi starvation triggers the homeostatic mechanism known as “Pi starvation responses” (PSR), which leads to induction of MYB transcription factors (MYB-TF) called Phosphate Starvation Regulator 1 (PHR1) and PHR1-like proteins. The increased levels of these proteins are related with an upregulation of miR399 and miR827. These miRNAs repress phosphate 2 (PHO2) and Nitrogen Limitation Adaptation (NLA), respectively. Both proteins are involved in the repression of phosphate transporter 1 (PHT1), and PHO2 also repress PHO1. Repression of NLA and PHO2 leads to PHT1 and PHO1 accumulation and consequently increase the Pi uptake as well as the accumulation of Pi in shoots.

The miRNAs miR399 and miR827 are important players in PSR since they repress genes involved in the repression of PHT1 and PHO1, contributing to their accumulation during stressed periods (**Figure 2**). The miRNA family miR399 (a–f) was the first one to be associated to Pi deficiency status. Members of the family are usually upregulated in response to the stress (Fujii et al., 2005) and when overexpressed by transgenes in *Arabidopsis*, the translocation of Pi from roots to shoots is enhanced and consequently the accumulation of Pi in shoots are from five to six times higher than in wild type plants (Chiou et al., 2006). In *Solanum lycopersicum*, in addition to the aforementioned effect, transgenic plants overexpressing miR399 also exhibit increased elimination of acid phosphatase and protons through roots, facilitating hydrolysis of organic phosphorus in the soil (Gu et al., 2010). In spite of several genes been predicted as targets of miR399, only an ubiquitin binding enzyme E2 encoded by the *PHO2* gene was validated as being regulated by it (Allen et al., 2005). This is primarily a gene expressed in the plant vascular system and downregulated in Pi deficient states. Multiple miR399 target sites were identified in the 5′-UTR region of PHO2 (Aung et al., 2006; Bari et al., 2006; Chiou et al., 2006). As an E2 ligase, PHO2 mediates the ubiquitination of the Pi/H^+^ transporters during normal Pi conditions, preventing their trafficking to membranes (Huang et al., 2013; Park et al., 2014). PHO2 also mediates a post-translational inhibition of PHO1 (Liu et al., 2012). Therefore, by repressing PHO2, miR399 contributes to the accumulation of both PHT1 and PHO1 during Pi starvation. Interestingly, miR399 species act as mobile signals, as their biogenesis take place at the shoots (Lin et al., 2008; Pant et al., 2008). Since, miR399* are also detected in roots, the miRNA is probably loaded as dsRNA into the phloem sap and systemically transported to roots. The identification of miR399 sequences in phloem sap of Pi-starved plants supports this model of action (Buhtz et al., 2008). On the other hand, miR399 family members can be regulated by the long non-coding gene Induced by Phosphate Starvation 1 (IPS1) in *Arabidopsis*. IPS1 has sequence complementary to miR399, but with unpaired nucleotides in the predicted cleavage site. It has been shown that IPS1 is not cleaved, but instead sequesters miR399 and block its action adding another level of regulation in PSR (Franco-Zorrilla et al., 2007).

Similar to miR399, miRNA, miR827 also repress a protein that is involved with PHT1 ubiquitination (Hsieh et al., 2009; Pant et al., 2009). Apart from being ubiquitinated during trafficking by PHO2, PHT1 may also suffer ubiquitination when already located in the plasma membrane by the Nitrogen Limitation Adaptation (NLA) protein, an E3 RING ubiquitin enzyme (Kant et al., 2011; Lin et al., 2013). NLA-mediated ubiquitination of PHT1 leads to endocytosis and degradation of the protein. Accordingly, a reduction of PHT1 endocytosis is observed in *nla* mutants (Lin et al., 2013). The post-transcriptional regulation of the NLA encoding gene by miR827, therefore, helps to stabilize PHT1 levels during stress conditions. Interestingly, *O. sativa* miR827 apparently also coordinate Pi stress responses, but by an independent pathway, since NLA homologues are not targeted by the miRNA in the plant (Lin et al., 2010; Wang et al., 2012).

Apart from miR399 and miR827, several other miRNA families have been associated to Pi responses (**Table 2**). Some of them are young clade-specific miRNAs, acting on specific molecular adaptations in response to Pi issues, while others are highly conserved (Hsieh et al., 2009; Pant et al., 2009; Gu et al., 2010; Lin et al., 2010; Lundmark et al., 2010; Matts et al., 2010; Valdés-López et al., 2010; Zeng et al., 2010; Kuo and Chiou, 2011). In a particular species, some miRNA families are up or downregulated in one or more tissues. There are still some families which are exclusively expressed in one plant tissue. This spatial limited activation of miRNAs is related to particular ways in which these areas organize their responses to P deficiency. In *Lupinus albus*, 35 miRNAs families were identified as differentially expressed; roots, stems, and leaves had, respectively, 24, 15, and 22 miRNAs families up or downregulated, demonstrating the possible overlapping nature of the response (Zhu et al., 2010). However, most of the conserved miRNAs misregulated under PSR are also responsive to other types of stresses, including nutrient deprivation. Their functions are therefore probably associated to general responses triggered in different types of stress conditions.

**Table 2:** P-responsive miRNAs with respective expression profiles in different tissues and species during P-starvation.

Plant species	miRNAs	Expression profile	Tissue	Reference
*Arabidopsis thaliana*	miR156, miR399, miR778, miR827, miR2111-5p, miR2111-3p	Upregulated	Root	Hsieh et al. (2009), Pant et al. (2009), Lundmark et al. (2010)
	miR163, miR399, miR778, miR827, miR828, miR2111-5p, miR2111-3p	Upregulated	Stem	
	miR169, miR395, miR398, miR402	Downregulated	Root, stem	
	miR399, miR447, miR778, miR827, miR2111-5p, miR2111-3p	Upregulated	Seedling	
	miR169, miR398	Downregulated		
*Glycine max*	miR159	Upregulated	Root	Zeng et al. (2010)
	miR166, miR319, miR398	Downregulated		
*Lupinus albus*	miR156, miR159, miR160, miR164, miR166, miR167, miR168, miR319, miR396, miR437, miR809, miR830, miR845, miR857, miR895, miR896, miR1222	Upregulated	Root	Zhu et al. (2010)
	miR168, miR171, miR395, miR399, miR447, miR477, miR818, miR863, miR866, miR903	Downregulated		
	miR171, miR395, miR447, miR472, miR818, miR854, miR866, miR903, miR904	Upregulated	Stem	
	miR159, miR164, miR166, miR319, miR857, miR895	Downregulated		
	miR168, miR171, miR395, miR399, miR447, miR477, miR818, miR863, miR866, miR903	Upregulated	Leaf	
	miR156, miR159, miR160,miR164, miR166, miR167, miR396, miR397, miR530, miR830, miR857, miR896	Downregulated		
*Medicago trunculata*	miR5229a,b, miR5206, miR160f, miR5205, miR169d,l, miR169d,e.2,l,m, miR160c, miR171h, miR167, miR5244, miR5232, miR5281b-f, miR5250, miR2086, miR166b.2,c.2,f-2, miR396b, miR5213, miR162	Upregulated	(AM related)	Devers et al. (2011)
	miR4414a, miR5285a-c	Downregulated		
*Orysa sativa*	miR399, miR827	Upregulated	Root, stem	Zhou et al. (2008), Lin et al. (2010)
*Panicum virgatum*	miR399	Upregulated	Seedling	Matts et al. (2010)
*Phaseolus vulgaris*	miR399	Upregulated	Root, leaf	Valdés-López et al. (2010)
	miR157	Upregulated	Nodules	
	miR397, miR398	Downregulated	Leaf	
*Solanum lycopersicum*	miR319, miR394, miR399	Upregulated	Root	Chiou et al. (2006), Gu et al. (2010)
	miR399	Upregulated	Stem, leaf	
	miR158, miR862	Downregulated	Root	Gu et al. (2010)
	miR158, miR169g, miR172, miR172b, miR319, miR398, miR771, miR775, miR837	Downregulated	Leaf	

### miRNA, Phosphorus, and Mycorrhiza Symbiosis

One of the most widespread adaptations to P deficiency is the interaction with arbuscular mycorrhizal fungi. In this interaction, fungi promote a more efficient uptake of water and nutrients (P among them) by roots and simultaneously receive carbohydrates required for their metabolism (Delaux et al., 2013).

microRNAs are one of the mechanisms used by plants in the regulation of this symbiosis. In the model species *M. truncatula*, several miRNAs have already been predicted as participants in this physiological response (Devers et al., 2011). The miR171h operates in the spatial regulation of fungi root colonization by targeting the gene encoding Nodulation Signaling Pathway 2 (NSP2), required for the production of stimulatory plant hormones (Lauressergues et al., 2012). The miR396, which exerts control over several transcription factors related to root development, undertakes a repressive action in mycorrhizal colonization in this species. Mutants overexpressing this miRNA were less colonized than control plants, while those that inactivated the same miRNA were significantly more colonized by AM fungi (Bazin et al., 2013). In *M. truncatula* and *Nicotiana tabacum* miR399 was also overaccumulated in mycorrhizal tissues in comparison to non-mycorrhizal ones indicating a possible role in this interaction (Branscheid et al., 2010). The miR5229, whose expression is detected only in mycorrhizal cells, has a Heme peroxidase as a potential target (Devers et al., 2011). The miR169, which targets the TF MtHAP2-1, a member of the CCAAT-binding family, was reported to be expressed in mycorrhizal roots (Combier et al., 2006). Additionally, miR160c was demonstrated to be another miRNA induced in mycorrhizal tissues, while miR5204, was detected to localize among the arbuscules as well as to be phosphate responsive (Devers et al., 2011).

## Potassium and Plant miRNAs

Potassium (K^+^) is an essential plant macronutrient involved in several signaling pathways. It is important for the metabolic adjustment during plant development and reproduction, yield and responses to salinity, drought, cold, high light, and hormones (Bose et al., 2014a,b,c; Demidchik et al., 2014; Straltsova et al., 2014; Zorb et al., 2014; Daras et al., 2015; Zhang et al., 2015). Each plant species has its physiological mechanism to the uptake of K^+^. The efficiency of the uptake and physiological role of K^+^ has to be considered when improving crop yield, plant tolerance to biotic and abiotic stresses (Zorb et al., 2014). A diverse set of genes coding for K^+^ channels and ion transporters has been functionally characterized to unravel the molecular physiology of K^+^ in plants (Schroeder et al., 1994; Chen et al., 2008; Adams and Shin, 2014; Cherel et al., 2014). Genes related to the homeostasis of potassium in plants belong to classes of AKT1 and KT/KUP/HAK types, which code for ion channels and ion transporters, respectively (Adams and Shin, 2014). In cell membranes, K^+^ channels have different physiological roles depending on where they are functional. If expressed in guard cells, they function in the net influx of K^+^ and, if expressed in root cells, they function in the low and high affinity uptake of K^+^ (Schachtman, 2000). This differential expression reflects a crucial role of K^+^ channels in the uptake and water balance, osmotic potential and, in transpiration mechanisms. Also, transport proteins underlie long distance – from root xylem to the shoots – and intracellular movement of K^+^ (Adams and Shin, 2014). Under low amounts readily available in the soil, plants have to trigger a bunch of complex molecular mechanisms to fine tune the absorption, transport, and efficient use of K^+^ (Cherel et al., 2014).

**FIGURE 3:**
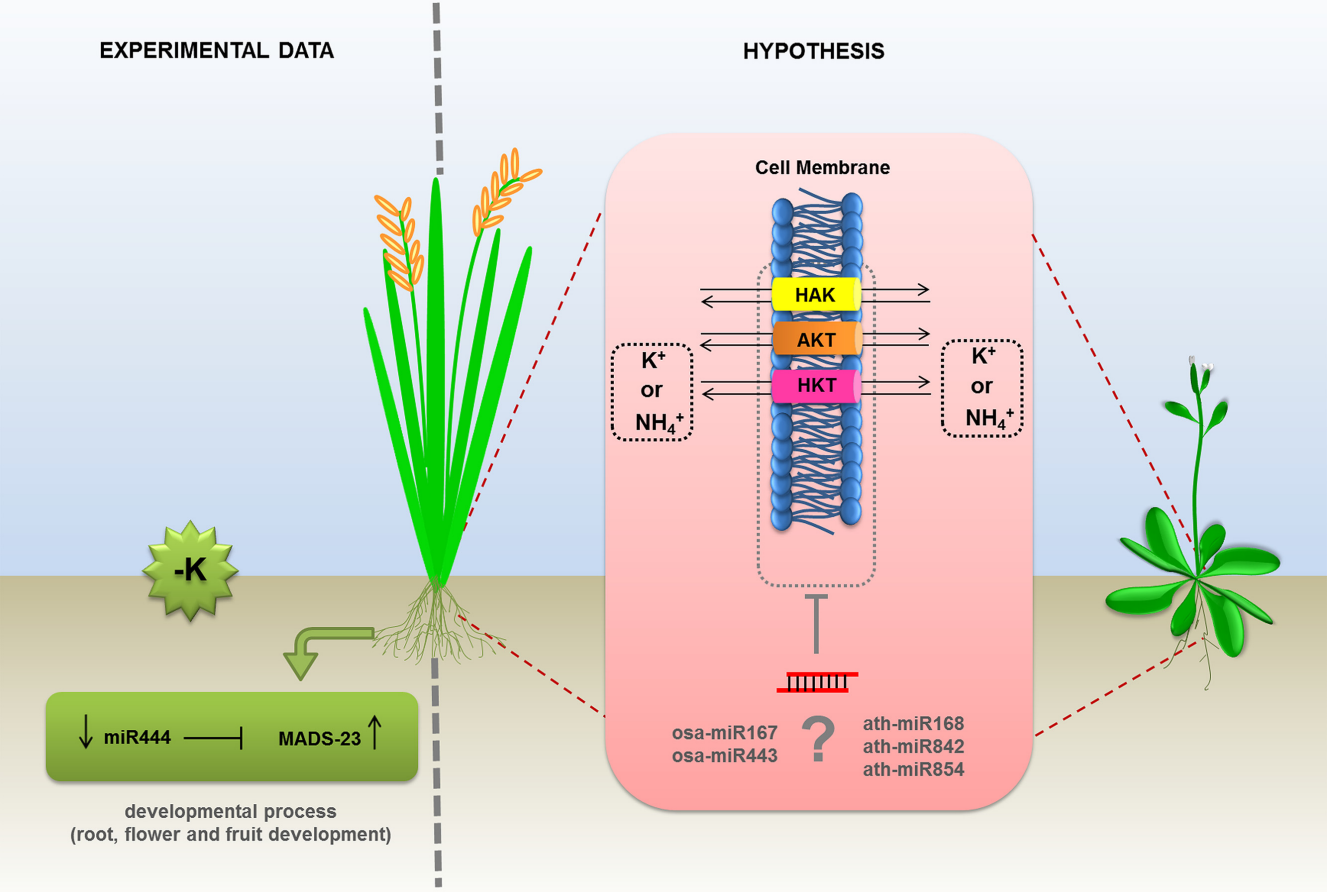
**Proposed models of regulation of potassium transportation by miRNAs in plants**. The expression levels of MADS-23 transcription factor, involved in potassium uptake, was experimentally demonstrated as regulated by miR444 in monocots. The transcripts of AKT (ankyrin potassium transporter), HAK, and HKT1 (high affinity potassium transporters) were predicted as putative targets of (?) miR167 and miR443 in rice and miR168, miR842, and miR854 in *Arabidopsis*. The direct effect of miRNAs on the mRNA levels of the members of potassium transporters inhibited by NH_4_^+^, is an hypothesis based on *in silico* analysis and remains to be confirmed.

In plants, miRNAs are also known to be involved a wide range of fine-tuned controls during development and in response to a variety of stresses (de Lima et al., 2012). In humans, a few reports reveal the regulatory role of miRNAs and potassium channel genes in the physiology of heart and lung (Li et al., 2013, 2014; Tatro et al., 2013). However, it is far from clear how miRNAs are directly affected by the uptake and physiology of potassium in plants. So far, the unique miRNA investigated with respect to K^+^ signaling was the monocots specific miR444a. In the work performed by Yan et al. (2014) miR444 was deeply evaluated about its involvement in N and Pi accumulation. However, the authors also explored the expression profile of this miRNA and its respective targets (MADS-23, MADS-27a, MADS-27b, and MADS-57) during K^+^ deprivation in rice roots. This condition caused a slightly decrease of miR444a levels. MADS-23 target was strongly induced compared to the control situation (**Figure 3**). MADS-box genes encode a family of transcription factors and are associated with several developmental regulatory pathways, from root to flower and fruit development (Becker and Theiβen, 2003). Other possible candidate to be investigated is the plant conserved miRNA miR167 which putatively targets ion transporters and genes coding for ion channel proteins (Griffiths-Jones et al., 2006; Zhang et al., 2013). In induced leaf senescence of rice plants, miR167a-3p putatively targets intracellular trafficking and vesicular transport genes (Xu et al., 2014). Another interesting hypothesis emerges with the competition of NH4^+^ and K^+^ for protein transporters in barley and *Arabidopsis* (ten Hoopen et al., 2010) and the possibility of miRNA regulation. The presence of NH4^+^ in potassium containing medium favors nitrogen uptake by HAK5 protein, which is suggested to be a high affinity K^+^ transporter (ten Hoopen et al., 2010). Under this nutrition circumstance, the protein AKT1 turns to be the main K^+^ uptake protein even in low K^+^ concentrations. Additionally, *in silico* tools can point to some K^+^ transporters proteins, like AKT, HAK, and HKT, as potential target sites for miRNAs in monocots and dicots species. Based on these findings, a dual ion uptake and a possible miRNA based-regulation point to a fine tuned physiological role of ion transport in plants (**Figure 3**, right). Undoubtedly, miRNAs have important physiological roles in plants under abiotic conditions and nutrient availability in soils. However, the targeting of potassium transporter genes by miRNAs remains to be validated and the dual ion uptake mediated by miRNAs remains to be investigated in detail.

A range of previous works discussed the counteraction between miRNAs and putative ion transporters and genes coding for proteins that are potentially potassium dependent, as well as other proteins and miRNAs that have a convincing role in plant nutrition (Pant et al., 2009; Buhtz et al., 2010; Kehr, 2013). Additionally, targeted genes are evaluated based on miRNA ability to chop mRNAs. Although it has been proposed that translation inhibition is not a major miRNA regulatory mechanism in plants, the analysis of protein levels could confirm those ion transporters and other miRNA targets that were based on bioinformatics approaches (Chen, 2004; Voinnet, 2009).

Differential regulation and functions of K^+^ responsive genes are widely cited in the literature (Cherel et al., 2014). High throughput analyses expand the view that ion transporters and protein channels are putatively regulated by a series of miRNAs. Although it still has to be extensively and experimentally confirmed, it is suggested that miRNAs play a pivotal role in the regulation of genes coding for proteins that sense, uptake and transport K^+^ in plants.

## Concluding Remarks and Future Perspectives

It has been very well documented that environmental changes can affect miRNA levels in plants. In this context, it would be expected that nutrient availability should also affect miRNA levels and homeostasis. In this review, we described a series of studies demonstrating how the macronutrients N, P, and K can affect miRNAs levels, triggering their increase or decrease, with an associated effect on the expression of different target genes. Both, excess or low level of nutrients can be sensed by plants as and abiotic stress. This will trigger a series of common mechanisms or specific strategies to face an emerging stress situation. Since the role of a miRNA is directly associated with its target function, we could observe that the majority of miRNAs involved in NPK deprivation are associated with mechanisms involved in the adaption to stress conditions. Actually, it was difficult to clarify about how the miRNAs are involved in K^+^ metabolism, once the literature is still scarce to help to understand the molecular basis of miRNA/K^+^ interaction in plants. However, based on the hypothesis that miR444 is involved in the K^+^ signaling, we can suppose that a control of root development can also be involved in K^+^ metabolism. For N and P pathways in plants, the miRNAs roles have been extensively explored, and several routes were already very well characterized. In general, N and P starvation, affect miRNAs that will regulate genes involved in the uptake or reallocation of these nutrients. At the morphological and physiological level, miRNAs were proved to affect root architecture (suppressing primary root elongation, increasing lateral root), controlling NO_3_^-^ or Pi transporters, controlling shoot growth, affecting vegetative phase transition, and managing these nutrients leakage (**Figures 1** and **2**). Since some miRNAs are critical in NPK metabolism control and are related to plant adaption during these nutrients stresses, we must consider their potential use in plant genetic breeding.

So far, we looked for miRNAs that were commonly involved in the three nutrients routes, but no report was detailing a route involving a miRNA regulation for NPK. A recent work analyzed the miR444 expression during N, P, or K depletion (Yan et al., 2014). Changes in this miRNA was observed for all three nutrients treatment, however, the mechanism behind this behavior were just explored for N metabolism. In this way, we observed that there is a vast unexplored field which can address the regulation of miRNAs when plants are exposed to more than one nutrient stress. Another important consideration about miRNA and NPK signaling is that analyzing the works which describe miRNAs expression according NPK availability, we observe that there is a gap about how these miRNAs are controlled. So, to find out what genes are activating or repressing these miRNAs, could help to complete the regulatory network involved in the sensing of plant nutrients.

Despite the quite large amount of data that can be found in the literature, it is clear that there is no standardization among experiments carried out with distinct nutrients by different research groups. These variations are much higher when experiments were performed with different plant species. As effective variables found in experiments, we observed: the developmental stage of the plants, the tissues types, the time kinetics of the stress, the nutrient concentrations to trigger the stress, and the methods to evaluate the effects on miRNA and target gene expressions. An effort should be made by the plant community of physiologists, biochemists, and molecular biologists in order to create models and patterns to evaluate in a more universal way the effects of nutrients and other agents in miRNAs and target gene expression.

## Conflict of Interest Statement

The authors declare that the research was conducted in the absence of any commercial or financial relationships that could be construed as a potential conflict of interest.
